# Congenital Visceral Leishmaniasis

**DOI:** 10.3201/eid1202.050449d

**Published:** 2006-02

**Authors:** Catharina C. Boehme, Ulrike Hain, Astrid Novosel, Susanna Eichenlaub, Erna Fleischmann, Thomas Löscher

**Affiliations:** * University of Munich, Munich, Germany

**Keywords:** congenital visceral leishmaniasis, kala-azar, immunoblot, PCR, letter

**To the Editor:** Visceral leishmaniasis (VL) is usually transmitted by phlebotomine sandflies. Nonvector transmission occasionally occurs through blood transfusions, contaminated needles of drug users, organ transplants, or laboratory infection (*1*). Only a few cases of congenital transmission have been reported. We describe a case of VL in a German infant, who never had been to a VL-endemic area. Most likely, the parasite was congenitally transmitted from the asymptomatic mother to her child.

A 9-month-old girl had a 4-week history of intermittent fever, recurrent upper respiratory tract infections, and failure to thrive. Physical examination showed a distressed infant with bilaterally enlarged cervical lymph nodes, hepatosplenomegaly, and a rectal temperature of 40°C. The following laboratory results were remarkable: hemoglobin 6.4 mg/dL, erythrocyte count 3.3 million/μL with 10.9% reticulocytes, platelet count 74,000/μL, and leukocyte count 4,300/μL (29.8% neutrophils, 62.3% lymphocytes, 7.4% monocytes, 0.5% basophils, and 0% eosinophils). Serum electrophoresis showed pronounced hypoalbuminemia and hypergammaglobulinemia. Abdominal sonography verified hepatosplenomegaly. Cultures from blood and other materials as well as additional investigations for a wide spectrum of infectious diseases, including HIV infection, were negative. Leukemia was suspected, and a bone marrow biopsy was performed. It showed enhanced myelo-, erythro-, and thrombopoesis with slight lymphopenia but no leukemic cells. However, *Leishmania* amastigotes were detected in bone marrow macrophages at a density of ≈1 to 2 parasitized macrophages per 400× oil immersion field, corresponding to a Chulay score of 1+ (*2*). Serology was positive for *Leishmania* spp. by indirect immunofluorescence antibody test, with cultured promastigotes of *L. donovani* used as antigen (immunoglobulin G [IgG] antibody titer 1:1,024). Specific antibodies against 14- and 16-kDa proteins of *L. infantum* promastigotes (Figure) were confirmed by immunoblot (*3*). Polymerase chain reaction (PCR) on scrapings of stained bone marrow slides amplified a *Leishmania* spp.–specific sequence of the internal transcribed spacer-1 gene (*4*), and subsequent *Hae*III-restriction fragment length polymorphism helped identify the species as *L. infantum* (Figure). Liposomal amphotericin B, at a daily dose of 4 mg per kg body weight, was given by infusion on 6 consecutive days and repeated on days 14 and 21. The therapy was well tolerated. Within 3 days, the fever subsided. The child recovered completely, and blood cell counts reached normal values 5 weeks after treatment was begun.

**Figure:**
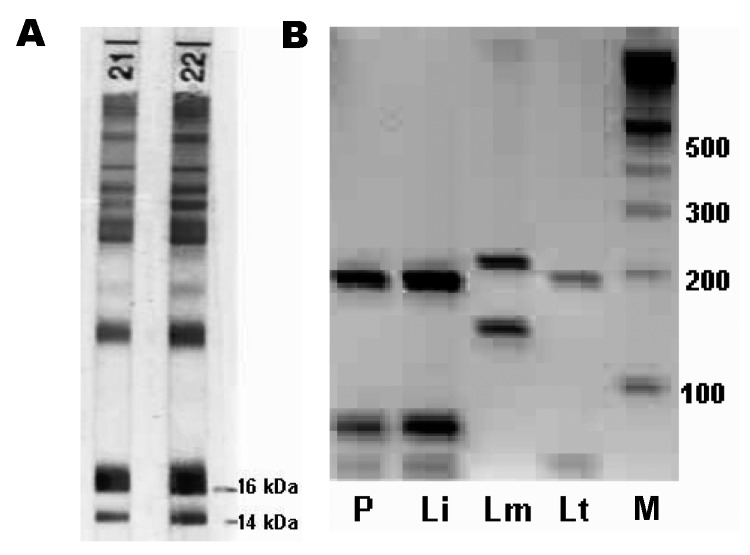
A) Immunoblot of the patient (strip no. 21) and the patient's mother (strip no. 22) showing specific antibodies against 14- and 16-kDa proteins of *Leishmania infantum*. B) restriction fragment length polymorphism patterns after *Hae*III digestion of the ribosomal internal transcribed spacer 1 polymerase chain reaction products. P, patient; Li, *L. infantum*; Lm, *L. major*; Lt, *L. tropica*; M, 100-bp ladder.

Since the child had never been outside Germany, vector transmission seemed highly improbable. The girl was born to a 26-year-old prima gravida, prima para, woman at 39 weeks' gestation by spontaneous labor; the infant's birth weight was 3,350 g, and she was 51 cm long. She showed normal development until the age of 8 months.

The mother had been healthy during pregnancy and had no history of serious disease; she did not show any pathologic findings at clinical investigation or in standard laboratory tests. However, *Leishmania* serologic tests conducted on blood samples from the mother showed positive results (IgG antibody titer 1:128 against promastigotes of *L. donovani*), and immunoblot analysis confirmed specific antibodies (Figure). During the last 15 years, she had spent holidays every year in Spain (Alicante) but had never been to a tropical country. She stayed in Spain during weeks 29–32 of her pregnancy. However, she could not remember any episodes of fever. She was not addicted to drugs nor had she ever received any blood products. Microscopic and PCR examinations of the mother's blood (buffy coat) and breast milk were performed with negative results. Cultures of both specimens in NNN medium were also negative. Since she was asymptomatic, a bone marrow biopsy was unwarranted. Four months later, she became pregnant again. No abnormalities were noted during pregnancy, delivery, or development of the second child.

Although sandflies (*Phlebotomus mascittii* Grassi, 1908) were recently found for the first time at 3 different locations along the upper Rhine Valley in southwestern Germany (*5*), no evidence exists for autochthonous transmission of leishmaniasis in Germany. Congenital transmission from the infected but asymptomatic mother is the most probable scenario in our case. Since 1926, only 10 case reports of congenitally acquired VL have been published (reviewed in 6). Most cases have been observed after the mother had VL during pregnancy. One previous report describes congenital transmission from an asymptomatic mother to her child (*6*). However, this rarity in reporting does not necessarily reflect the frequency of the event. In VL-endemic areas, cases of congenital VL cannot be distinguished from cases of infection by vector transmission during the first year of life. Congenital transmission may occur either through blood exchange from the mother to the child during labor or by transplacental infection during pregnancy. Which of the 2 transmission routes led to infection in our case is unclear. In the congenital cases reported to date, typical symptoms of the disease developed from 4 weeks to 18 months (mean 8.5 months) after birth. The incubation period after vector transmission is also highly variable (typically 2–6 months but varying from 10 days to >10 years [1]). All patients reported have been treated with pentavalent antimonial agents; this treatment is still widely used in VL-endemic areas, but it has considerable side effects, and resistance is increasing (*1*). Liposomal amphotericin B is the drug of choice for treating Mediterranean VL. An alternative, especially for low-income countries, is oral treatment with miltefosine.

This report suggests that in infants with fever, splenomegaly, and pancytopenia, VL should be considered even if the patient has not been to an disease-endemic area. Congenital transmission is possible, not only as a consequence of VL during pregnancy but also by transmission from an asymptomatic mother to her child in utero or during labor.
